# Brain structural and functional connectivity alterations are associated with fatigue in neuromyelitis optica spectrum disorder

**DOI:** 10.1186/s12883-022-02757-4

**Published:** 2022-06-27

**Authors:** Ying Zhang, Hong-xi Chen, Zi-yan Shi, Qin Du, Jian-cheng Wang, Xiao-fei Wang, Yu-han Qiu, Yan-lin Lang, Ling-yao Kong, Lin-jun Cai, Xue Lin, Zi-chao Mou, Wen-qin Luo, Shuang-jie Li, Hong-yu Zhou

**Affiliations:** Department of Neurology, West China Hospital, Sichuan University, Guo Xuexiang #37, Chengdu, 610041 China

**Keywords:** Neuromyelitis optica spectrum disorder, Fatigue, Pallidum, Nucleus accumbens, Cerebellum

## Abstract

**Background:**

Many patients with neurological disorders experience chronic fatigue, but the neural mechanisms involved are unclear.

**Objective:**

Here we investigated whether the brain structural and functional connectivity alterations were involved in fatigue related to neuromyelitis optica spectrum disorder (NMOSD).

**Methods:**

This prospective pilot study used structural and resting-state functional brain magnetic resonance imaging to compare total cortical thickness, cortical surface area, deep gray matter volume and functional connectivity (FC) between 33 patients with NMOSD and 20 healthy controls (HCs). Patients were subgrouped as low fatigue (LF) and high fatigue (HF).

**Results:**

HF patients scored higher on the Hamilton Anxiety Rating Scale and Hamilton Rating Scale for Depression than LF patients and HCs. The two patient subgroups and HC group did not differ significantly in cortical thickness, cortical surface area and volumes of the bilateral caudate nucleus, bilateral putamen, bilateral amygdala, bilateral hippocampus, bilateral thalamus proper or right nucleus accumbens (*p* > 0.05). However, after correcting for age, sex, years of education, anxiety and depression, HF patients showed larger left pallidum than HCs (0.1573 ± 0.0214 vs 0.1372 ± 0.0145, *p* = 0.009). Meanwhile, both LF patients (0.0377 ± 0.0052 vs 0.0417 ± 0.0052, *p* = 0.009) and HF patients (0.0361 ± 0.0071 vs 0.0417 ± 0.0052, *p* = 0.013) showed smaller left nucleus accumbens than HCs.. Compared with LF patients, HF patients showed significantly decreased FC between the left pallidum and bilateral cerebellar posterior lobes.

**Conclusions:**

This was the first evidence linking structural and functional alterations in the brain to fatigue in NMOSD, and in the future, long term follow-up was necessary.

## Introduction

Neuromyelitis optica spectrum disorder (NMOSD) is an autoimmune disorder affecting the central nervous system and associated with the presence of aquaporin-4 immunoglobulin G antibodies (AQP4-IgG) [1]. Up to 70% of patients with NMOSD experience fatigue [2, 3], which can significantly reduce health-related quality of life [3, 4]. Such fatigue has also been reported in a high percentage of patients with multiple sclerosis (MS), another autoimmune disease affecting the central nervous system. Indeed, several pathophysiological mechanisms have been proposed at the origin of fatigue in MS, and mainly attributed to atrophy of brain gray matter [5, 6]. Additionally, some other explanations may also contribute to the development of MS-related fatigue, such as damage to fronto-striatal and temporo-insular, alterations of frontoparietal β-adenosine triphosphate, impaired interactions between functionally related cortical and subcortical areas [7–9].

However, what causes fatigue in NMOSD is unclear. Central location or number of segments of spinal cord injury does not appear to be the cause [10]. Actually, in neurological disorders, defects in certain brain regions may be at fault, such as in pathways interconnecting the basal ganglia, thalamus, and higher cortical centers, and pathways involved in hypothalamic-pituitary-diencephalic syndrome [11].

Therefore, we hypothesized that brain changes may be related to fatigue in patients with NMOSD. To explore this hypothesis, we used magnetic resonance imaging (MRI) to compare structural and functional connectivity alterations of potentially important brain regions between NMOSD patients who experience low fatigue or high fatigue, and healthy controls.

## Methods

This study was approved by the Medical Ethics Committee of West China Hospital of Sichuan University, and was performed in accordance with relevant guidelines and regulations. Written informed consent was obtained from all subjects.

### Participants

From August 2016 to February 2021, patients with NMOSD [1] were consecutively recruited into our study; in parallel, we recruited 20 healthy controls (HCs) from the community, whom we matched to the patients based on age, sex and years of education. We excluded patients who had had an acute attack fewer than 3 months before the study, who were seronegative for AQP4-IgG based on flow cytometry of serum [12], who had psychiatric or other neurological disorders, who had visible brain lesions on conventional MRI, or for whom MRI was contraindicated.

### Clinical assessment

Demographic and clinical data were collected. Patients were assessed the severity of anxiety, depression and disability using the Hamilton Anxiety Rating Scale (HAMA), Hamilton Rating Scale for Depression (HRSD) and Kurtzke Expanded Disability Status Scale (EDSS), respectively. Fatigue was assessed in patients using the Fatigue Impact Scale [13], which is widely used in patients with MS and NMOSD, including in China [4, 14–16]. The FIS provides self-report of the perceived impact of fatigue on cognitive (10 items), physical (10 items) and social (20 items) dimensions during the preceding month. For each item, subjects respond on a scale from 0 (no problem) to 4 (extreme problem). The scores on all individual items are summed to obtain a global score (maximum 160).

The median score (43) of FIS-global coming from NMOSD patients was set as the cut-off. Therefore, those NMOSD patients with a global FIS score ≤ 43 were categorized as low fatigue (LF) group, and those patients with a global FIS score > 43 were categorized as high fatigue (HF) group. Data were compared between each patient subgroup and the HCs.

### Brain image acquisition

Patients and HCs underwent imaging using the same 3.0-T scanner (MR750, General Electric, Fairfield, Connecticut, USA) at the University of Electronic Science and Technology of China. Padded clamps were used to minimize head motion, and subjects were asked to remain awake and motionless with their eyes closed during image acquisition. Structural MRI and resting-state functional MRI (rs-fMRI) were collected in the same scanning session. The structural images were scanned using a T1-weighted, three-dimensional, fast spoiled gradient recall echo sequence (repetition time 5.16 ms, echo time 1.7 ms, inversion time 450 ms, slice thickness 1 mm, voxel size 1 mm × 1 mm × 1 mm, field of view 256 mm × 256 mm, 256 × 256 matrix, flip angle 8°, and 192 axial slices). The rs-fMRI was performed with a gradient-echo echo-planar imaging sequence (repetition time 3000 ms, echo time 30 ms, slice thickness 3.0 mm, field of view 192 mm × 192 mm, matrix 64 × 64, flip angle 90°, 205 time points, and 50 slices).

### Brain image processing

#### Structural MRI processing

**Fig. 1:**
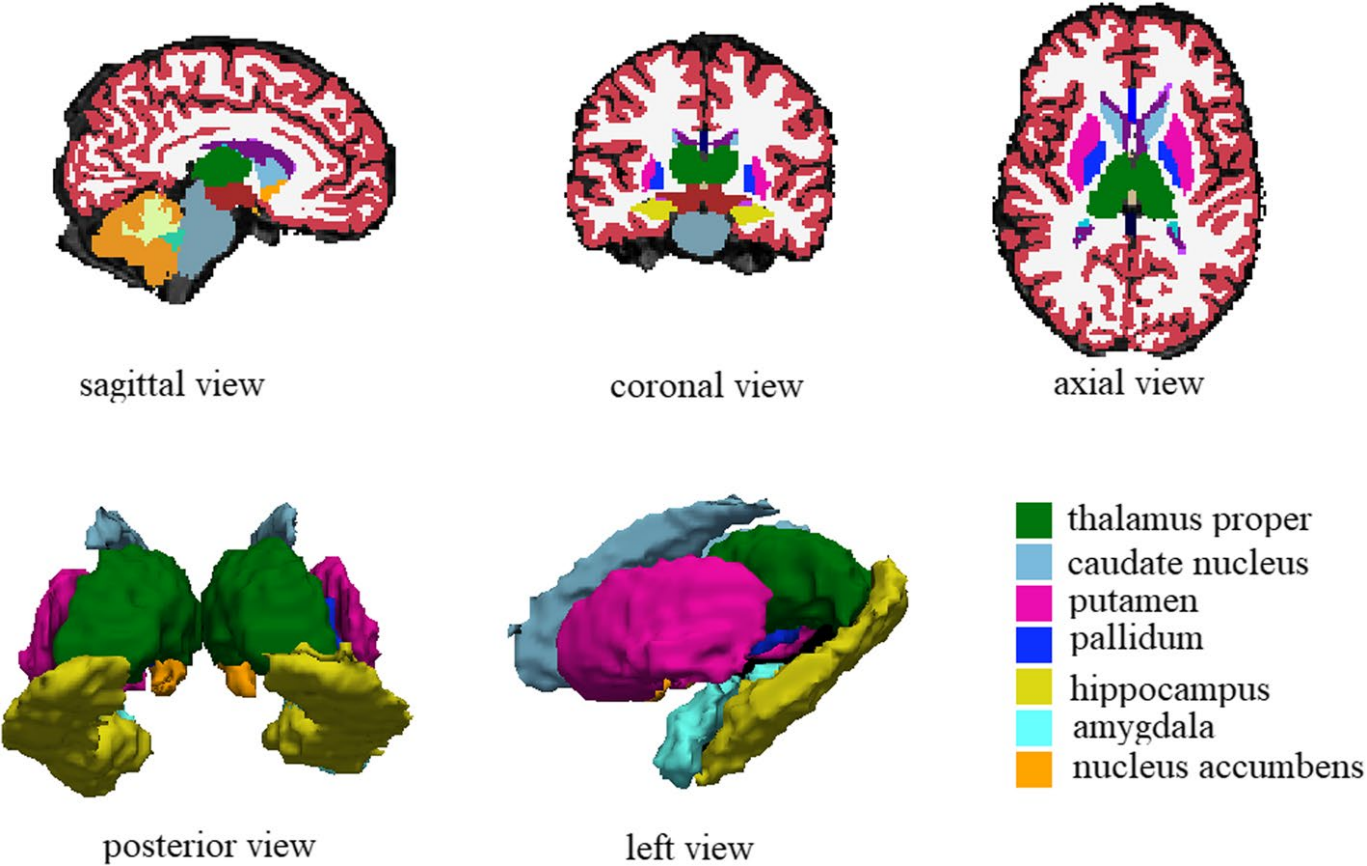
The segmentation of deep gray matter

Scans were checked by an investigator blinded to the clinical information, and subjects with visible brain lesions were excluded. Total cortical thickness, cortical surface area and volume of deep gray matter were measured using FreeSurfer 6.0.0 (http://surfer.nmr.mgh.harvard.edu/fswiki/FreeSurferWiki) as described [17]. Briefly, the automated recon-all pipeline, with default settings, was used to perform: 1) skull stripping and brain extraction, 2) corrections for motion, head shape and position, 3) Talairach transformations, 4) intensity normalization, 5) segmentation of subcortical white and gray matter, 6) smoothing, topology correction and surface deformation, and 7) cortical and subcortical parcellation. All processed images were checked for errors and corrections. For the asymmetry of brain [18, 19], total cortical thickness, cortical surface area and volume of deep gray matter were calculated separately for the left and right hemispheres. Cortical thickness was measured at each vertex as the distance between gray matter/white matter boundary and gray matter/ cerebrospinal fluid boundary. Cortical surface area was quantified by averaging the triangular size surrounding the tessellated cortical vertices of the pial surface. Estimates of volumes of deep gray matter, defined as basal ganglia (caudate nucleus, putamen and pallidum), amygdala, hippocampus, nucleus accumbens and thalamus proper (Fig. 1) were extracted. Next, volumes of these structures were corrected for head size by diving each value by the estimated intracranial volume (eTIV) provided by the Freesurfer pipeline and multiplying the result by 100 (structure volume / eTIV × 100).

#### Rs-fMRI processing

The preprocessing was performed using the Data Processing Assistant for Resting-State fMRI (DPARSF 4.3, http://rfmri.org/DPARSF), which is based on Statistical Parametric Mapping (SPM, http://www.fil.ion.ucl.ac.uk/spm) and the toolbox for Data Processing & Analysis of Brain Imaging (DPABI, http://rfmri.org/DPABI) [20]. The first 10 volumes were discarded. Preprocessing steps included data conversion, slice timing, realignment, nuisance regression, normalization, smoothing, and filtered at 0.01 to 0.1 Hz. The clusters with structural alteration in the former structural MRI processing were set as the seed regions of interest (ROIs) to calculate functional connectivity (FC). The seed-based FC was performed by calculating the temporal correlation between the ROIs and the rest of brain in a voxel-wise manner and transformed to the z-value images. Any subjects whose mean frame-wise displacement (FD_Jenkinson) > 0.2 mm were excluded.

### Statistical analyses

Results were presented as mean ± SD for normally distributed data, median [interquartile range (IQR)] for skewed data, and percentages for categorical data. Differences in demographic and clinical data were assessed for significance using STATA 14.1. Differences in normally distributed data were tested using analysis of variance, followed by post-hoc comparisons. Differences in skewed data were tested using the Kruskal–Wallis and Wilcoxon rank-sum tests. If the Kruskal–Wallis test showed differences among groups, the Kwallis2 test was used for pairwise comparisons. *P* < 0.05 were considered significant.

For comparisons of brain structure, cortical surfaces from each subject were registered to a standard template surface space, and maps were smoothed at a full width half-maximum of 10 mm. Differences in total cortical thickness and cortical surface area were investigated using a general linear model, with age, sex, years of education and scores on the HAMA and HRSD as covariates. Using STATA 14.1, differences in corrected volumes of deep gray matter (basal ganglia, amygdala, hippocampus, nucleus accumbens and thalamus proper) were assessed using analysis of variance, followed by post hoc pairwise comparisons using multiple linear regression, with age, sex, years of education and scores on the HAMA and HRSD as covariates. Potential correlations between corrected volumes of deep gray matter and clinical characteristics (EDSS score and disease duration) were explored using multiple linear regression, with age, sex, years of education and scores on the HAMA and HRSD as covariates. *P* < 0.05 were considered significant.

Using the DPABI software, the differences in the strength of FC correlation between the ROIs and other voxels in the brain were compared with ANOVA and post hoc analysis was performed for multiple comparisons using Bonferroni method, with FD_Jenkinson, age, sex, years of education and scores on the HAMA and HRSD as covariates. The threshold correction was performed using the Gaussian random field (GRF), with *p* < 0.001 at the voxel level and *p* < 0.017 (for post hoc comparisons) at the cluster level indicating a significant difference.

## Results

### Characteristics of subjects

Thirty-three Chinese Han NMOSD patients (44.6 ± 11.1 years old; female, 31) and 20 HCs (45.2 ± 9.5 years old; female, 20) were included in the analysis. Sixteen of NMOSD patients were classified as HF and 17 of NMOSD patients were classified as LF. The two patient subgroups of NMOSD patients and the HCs did not differ significantly in age (*p* = 0.404), sex (*p* = 0.111) or years of education (*p* = 0.467). The two NMOSD patient subgroups did not differ significantly in median EDSS score (*p* = 0.637) or disease duration (*p* = 0.081). In contrast, HF showed more severe anxiety (as measured on the HAMA scale) and depression (as measured on the HRSD scale) than LF patients (*p* < 0.05). (Table 1).

**Table 1:** Comparisons of demographic and clinical characteristics between each patient subgroup and healthy controls

Characteristic	Healthy controls (*n* = 20)	Low fatigue (*n* = 17)	High fatigue (*n* = 16)	p
Age, yr	45.2 ± 9.5	42.2 ± 11.8	47.1 ± 10.0	0.404
Female, n (%)	20(100%)	15(88.2%)	16(100%)	0.111
Years of education	9.0 ± 2.7	9.9 ± 3.5	8.6 ± 3.3	0.467
EDSS score	–	3.5 (1.5,4)	3.3 (2.3,4)	0.637
Disease duration, yr	–	6.7 (4.3,9.8)	3.3 (2.4,7.3)	0.081
HAMA score	0 (0,2.5)	5 (2,7)	7.5 (4.5,12.5)	0.000
HRSD score	0 (0,1.5)	2 (1,7)	9.5 (5,14.5)	0.000
Global FIS score	0 (0,0)	20 (13,39)	71.5 (58,108.5)	0.000

Values are mean ± SD or median (interquartile range), unless otherwise noted

*yr* years, *EDSS* Expanded Disability Status, *HAMA* Hamilton Anxiety Rating Scale, *HRSD* Hamilton Rating Scale for Depression, *FIS* Fatigue Impact Scale

### Differences in total cortical thickness and cortical surface area

The two patient subgroups and HC group did not differ significantly in cortical thickness or cortical surface area (*p* > 0.05), regardless of whether the adjusted covariates were age, sex and years of education or were age, sex, years of education, and scores on HAMA and HRSD.

### Differences in deep gray matter volume

**Fig. 2:**
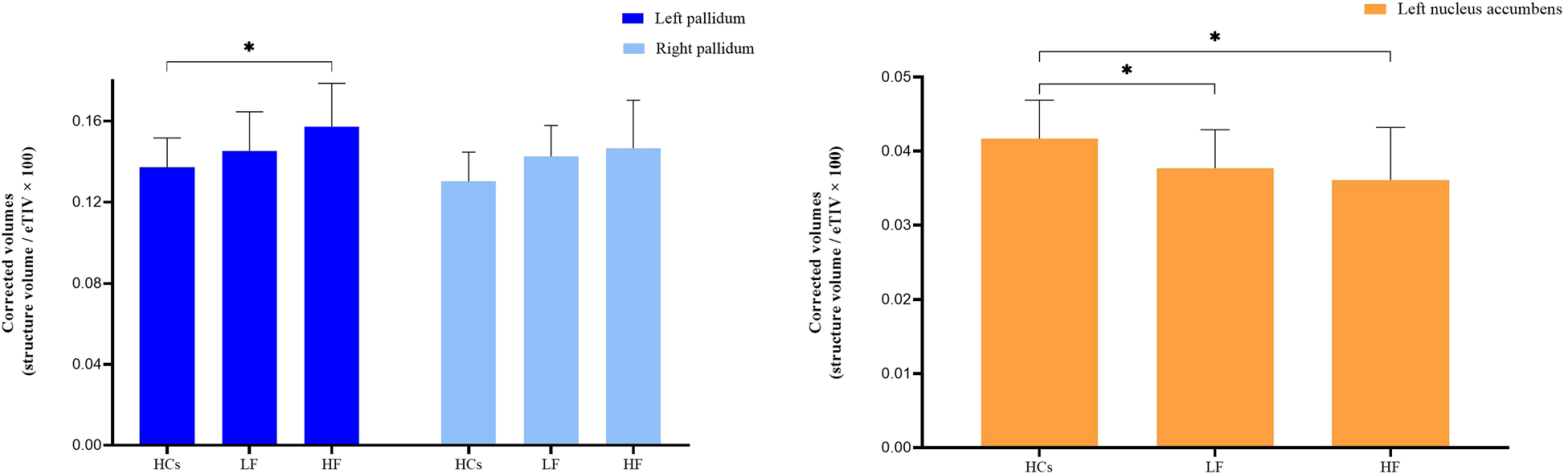
Corrected volumes (mean ± SD) of the bilateral pallidum and left nucleus accumbens between each patient subgroup and healthy controls. * indicating a significant difference adjusted for age, sex, years of education, anxiety and depression; eTIV, estimated intracranial volume; HCs, healthy controls; LF, low fatigue group; HF, high fatigue group

Using analysis of variance, corrected volumes of the bilateral pallidum and left nucleus accumbens showed significant differences between each patient subgroup and HCs (*p* < 0.05). (Table 2) After correcting for age, sex, years of education, and scores on the HAMA and HRSD, HF patients showed larger left pallidum than HCs (0.1573 ± 0.0214 vs 0.1372 ± 0.0145, *p* = 0.009). Meanwhile, both LF patients (0.0377 ± 0.0052 vs 0.0417 ± 0.0052, p = 0.009) and HF patients (0.0361 ± 0.0071 vs 0.0417 ± 0.0052, *p* = 0.013) showed smaller left nucleus accumbens than HCs. (Table 2, Fig. 2) However, the two patient subgroups and HC group did not differ significantly in corrected volumes of the bilateral caudate nucleus, bilateral putamen, bilateral amygdala, bilateral hippocampus, bilateral thalamus proper or right nucleus accumbens (*p* > 0.05). (Table 2).

**Table 2:** Comparisons of volumes of deep gray matter between each patient subgroup and healthy controls, corrected by the eTIV provided by the Freesurfer pipeline

Anatomical structures		Healthy controls	Low fatigue	High fatigue	*p* value
Caudate nucleus	Left	0.2435 ± 0.0230	0.2458 ± 0.0278	0.2441 ± 0.0161	0.951
	Right	0.2530 ± 0.0235	0.2556 ± 0.0304	0.2542 ± 0.0199	0.952
Putamen	Left	0.3562 ± 0.0271	0.3584 ± 0.0547	0.3550 ± 0.0481	0.974
	Right	0.3507 ± 0.0275	0.3669 ± 0.0489	0.3420 ± 0.0465	0.219
Pallidum	Left	0.1372 ± 0.0145	0.1454 ± 0.0193	0.1573 ± 0.0214	*0.008*
	Right	0.1302 ± 0.0145	0.1426 ± 0.0153	0.1466 ± 0.0238	*0.022*
Amygdala	Left	0.1105 ± 0.0131	0.1078 ± 0.0170	0.1103 ± 0.0125	0.829
	Right	0.1239 ± 0.0083	0.1209 ± 0.0169	0.1207 ± 0.0126	0.705
Hippocampus	Left	0.2868 ± 0.0224	0.2844 ± 0.0183	0.2848 ± 0.0245	0.935
	Right	0.2959 ± 0.0230	0.2932 ± 0.0209	0.2899 ± 0.0284	0.763
Nucleus accumbens	Left	0.0417 ± 0.0052	0.0377 ± 0.0052	0.0361 ± 0.0071	*0.017*
	Right	0.0372 ± 0.0033	0.0369 ± 0.0056	0.0357 ± 0.0053	0.625
Thalamus proper	Left	0.4974 ± 0.0376	0.5133 ± 0.0606	0.4814 ± 0.0581	0.225
	Right	0.4707 ± 0.0326	0.4840 ± 0.0395	0.4616 ± 0.0482	0.276

Values are mean ± SD of corrected volumes (structure volume / eTIV × 100). *eTIV* estimated intracranial volume

Additionally, among all patients with NMOSD, multiple linear regression showed that disease duration correlated negatively with corrected left pallidum volume (standardized coefficient = − 0.002, *p* = 0.016). Nevertheless, EDSS score did not correlate significantly with corrected volumes of the bilateral pallidum and left nucleus accumbens. (Table 3).

**Table 3:** Multiple linear regression to identify relationships between certain disease variables and corrected deep gray matter volumes in 33 patients with NMOSD

Variable	Left pallidum	Left nucleus accumbens	Right pallidum
EDSS	0.000, 0.942	0.001, 0.177	−0.000, 0.996
Disease duration	−0.002, 0.016	−0.000, 0.774	−0.001, 0.419

Values are shown as coefficients, followed by p value. The analysis was adjusted for age, sex, years of education, anxiety and depression. *NMOSD* neuromyelitis optica spectrum disorder

### FC results

**Fig. 3:**
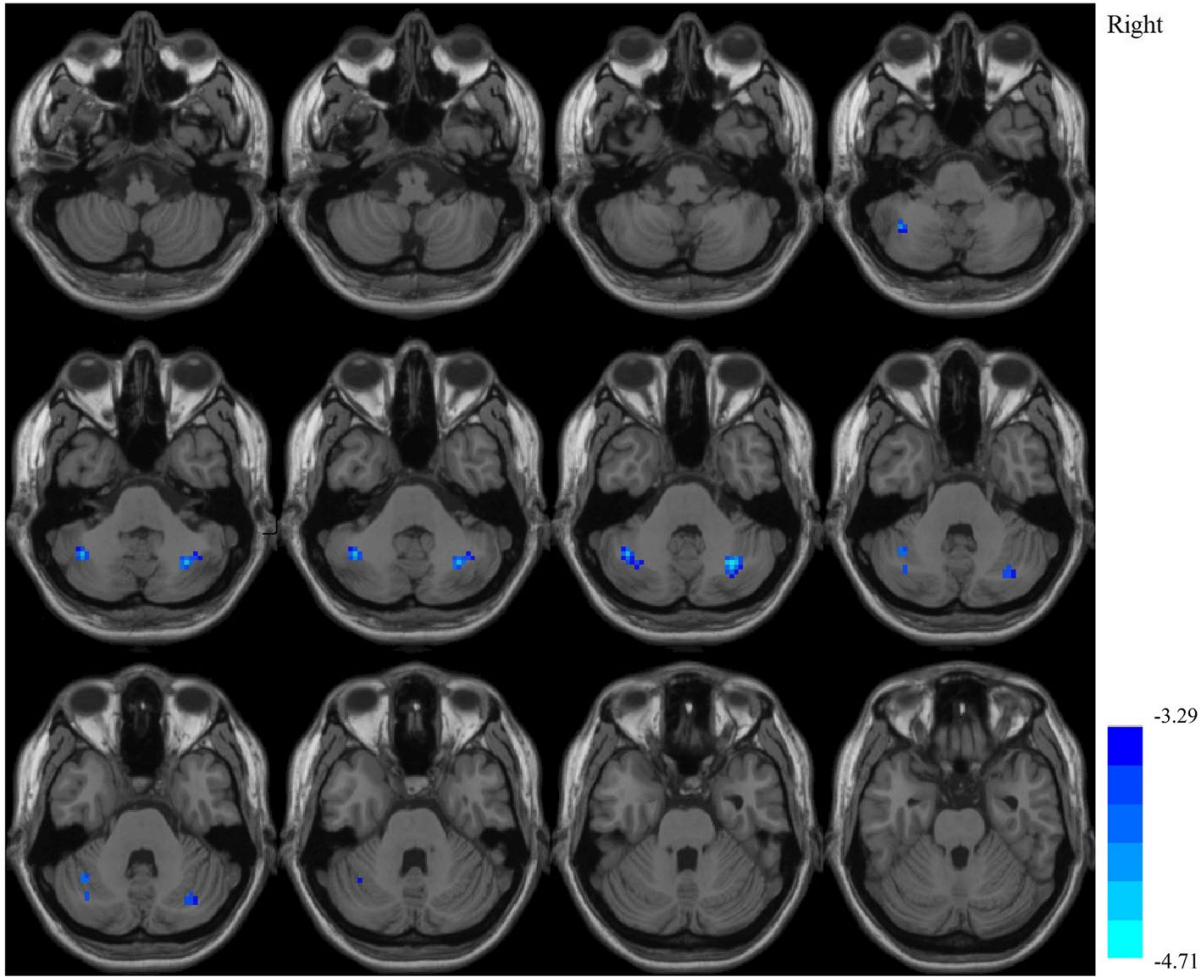
Decreased functional connectivity of NMOSD patients with high fatigue compared to patients with low fatigue. The left pallidum was set as the seeding region of interest. Covariates were FD_Jenkinson, age, sex, years of education and scores on the HAMA and HRSD. The color bar displayed Z-values. NMOSD, neuromyelitis optica spectrum disorder

The bilateral pallidum and left nucleus accumbens were set as the seed ROIs to calculate FC. However, between each patient subgroup and HCs, no regions showed significantly different FC between the right pallidum (*p* > 0.05) or left nucleus accumbens (*p* > 0.05) and the rest of brain. Meanwhile, different FC between the left pallidum and bilateral cerebellar posterior lobes was found between each patient subgroup and HCs (*p* < 0.05). In post hoc analysis, compared with LF patients, HF patients showed significantly decreased FC between the left pallidum and bilateral cerebellar posterior lobes, with or without corrected for age, sex, years of education and scores on the HAMA and HRSD (Table 4 and Fig. 3). However, compared with HCs, neither HF patients nor LF patients showed different FC between the left pallidum and the rest of brain.

**Table 4:** Brain regions with significantly decreased functional connectivity in NMOSD patients with high fatigue compared to patients with low fatigue

	Seed ROIs	Brain regions	Peak MNI			Cluster voxels	Peak z value
			X	Y	Z		
Analysis 1^*^	Left pallidum	Left cerebellum posterior lobe	−39	−57	−36	38	−4.5611
		Right cerebellum posterior lobe	30	−63	−36	42	−4.8523
Analysis 2^#^	Left pallidum	Left cerebellum posterior lobe	−39	−57	−36	33	−4.3456
		Right cerebellum posterior lobe	30	−63	−36	34	−4.7087

*NMOSD* neuromyelitis optica spectrum disorder, *ROIs* regions of interest, *MNI* Montreal Neurological Institute

^*^, with FD_Jenkinson as covariate; ^#^, with FD_Jenkinson, age, sex, years of education and scores on the HAMA and HRSD as covariates

## Discussion

Fatigue is an exhausting characteristic of neurological disorders, and disabling in NMOSD, MS and after stroke. Enhanced perception of effort and limited endurance of sustained physical and mental activities are the main characteristics of central fatigue [11]. The HF patients in our study showed more severe anxiety and depression than LF patients, consistent with previous studies linking fatigue, anxiety and depression in NMOSD [3, 21]. Therefore, when we compared brain structures in our study, we were careful to adjust for scores on the HAMA and HRSD as well as for demographic characteristics as potential confounders.

Seok, J. M., et al. found that atrophy in the right thalamus is strongly correlated with NMOSD-related fatigue severity [22]. It suggested that subcortical structures might serve as biomarkers of fatigue in NMOSD. Here we provided additional evidence from high-resolution structural brain MRI that changes in deep gray matter may be associated with fatigue in NMOSD. We found that HF patients had larger left pallidum than HCs, meanwhile, HCs showed larger left nucleus accumbens than both HF and LF patients. Additionally, the FC between the left pallidum and bilateral cerebellar posterior lobes decreased in HF patients compared with LF patients. These results may help guide research into the origins and, ultimately, management or prevention, of this potentially severe symptom of neurological disorders.

Our results are in line with previous studies, implicating that brain regional injury, such as basal ganglia and sensorimotor cortex, contributed to the pathogenesis of fatigue, not only in NMOSD, but also in MS, Parkinson’s disease and chronic fatigue syndrome. These alterations are thought to disrupt non-motor basal ganglia functions and striatocortical pathways [23–25]. In fact, fatigue in Parkinson’s disease has been associated with reduced serotonergic function in the basal ganglia, and increasing serotonin levels in the brain may treat such fatigue [26]. Furthermore, bilateral contemporaneous posteroventral pallidotomy for treatment of Parkinson’s disease is followed by fatigue, sleepiness,

changes in behavior, and poor initiative in executive functions despite improvement in motor control [27]. Therefore, we speculate that a circuit for fatigue might exist and bilateral pallidum may be part of this circuit. This circuit is associated with motivational regulation of voluntary activities and effort-related functions.

Usually, fatigue is associated with atrophy of basal ganglia and cortex in MS. [5, 6] However, in the present study, volumes of bilateral pallidum increased in HF patients and fatigue was not associated with brain atrophy like MS. Although both NMOSD and MS are autoimmune demyelinating diseases of the central nervous system, however, they have distinct immunological and pathological features [28]. In MS, degeneration occurs in the early disease course, and most patients evolves into a secondary progressive course. Even from the onset, some MS patients may present with a primary progressive course. However, in NMOSD patients, this progressive disease course is only rarely seen, and disability is tightly related to relapses [28]. These suggest that different pathological mechanisms participate in the formation of fatigue in NMOSD and MS, and more advanced functional imaging studies and in vivo studies are needed in order to understand the molecular basis of fatigue in NMOSD.

Along with pallidum, some other brain structures, such as amygdala, anterior cingulate.

cortex and nucleus accumbens, also are involved in the exertion of effort and effort-related choice behavior [29]. Nucleus accumbens is a critical component of brain dopamine systems. Previous studies suggest that the interaction between dopamine and adenosine in the nucleus accumbens contributes to regulating effort-related functions [29, 30]. Nevertheless, in the present study, we found that both HF and LF patients showed smaller left nucleus accumbens than HCs, however, HF patients did not show statistically significantly different volume of left nucleus accumbens compared with LF patients. This may attribute to the relatively small sample size. Further investigations are needed to determine the underlying mechanisms associated with fatigue in NMOSD.

In this study, the bilateral pallidum and left nucleus accumbens were set as the seed ROIs to calculate FC. Finally, we found the FC between the left pallidum and bilateral cerebellar posterior lobes decreased in HF patients compared with LF patients. Previous clinical and imaging studies have found that the cerebellum is engaged in cognitive and affective functions. Regions active during language, spatial processing and working memory tasks differ from those involved in motor control. Additionally, Stoodley, C. J., et al. found that the activation of cortices –cerebellar posterior lobe circuit engaged in cognition demanding tasks [31]. In MS, specific cerebellar lobules atrophy also plays a role in the development of fatigue [32]. Lower superior cerebellar peduncles volumes was found in MS patients with high fatigue [33]. And thalamic sub-region FC abnormalities with posterior lobes of the cerebellum also contribute to differentiating fatigued MS patients from non-fatigued MS patients [34]. Although the cerebellum’s primary function is to coordinate motor activity, extensive evidence suggests that the cerebellum may play an underappreciated role in producing the experience of fatigue [35], consistent with the present study.

Indeed, our study was not without limitations. First, the cross-sectional nature of our study prevented us from establishing causal relationships between changes in brain structure and fatigue in NMOSD. Longitudinal studies should explore this hypothesis. Second, in the present study, there was a trend towards higher disease duration in LF patients, although it was not statistically significant. Additionally, disease duration correlated negatively with left pallidum volume, which means that certain clinical characteristics may confound analyses of brain structure in the disorder. In the future, studies with larger sample size are essential to minimize the influence of confounding factors.

## Conclusion

Our data implicates that volumes of the bilateral pallidum and left nucleus accumbens are associated with fatigue in NMOSD, and the FC between the left pallidum and bilateral cerebellar posterior lobes decreased in HF patients. This prospective pilot study provides the first evidence linking structural and functional alterations in the brain to fatigue in NMOSD, which should be further explored in more advanced MRI longitudinal studies.

## Data Availability

The data that support the findings of this study are available from the corresponding author upon reasonable request.
